# PW02-018 - Impact of PSTPIP1 mutaions on clinical phenotype

**DOI:** 10.1186/1546-0096-11-S1-A158

**Published:** 2013-11-08

**Authors:** D Holzinger, P Lohse, S Faßl, J Austermann, T Vogl, W de Jager, S Holland, M Gattorno, C Rodriguez-Gallego, J Arostegui, S Fessatou, B Isidor, K Ito, H-J Epple, J Bernstein, M Jeng, G Lionetti, P Ong, C Hinze, B Sampson, C Sunderkoetter, D Foell, J Chae, A Ombrello, J Brady, I Aksentijevich, J Roth

**Affiliations:** 1 Institute of Immunology, University Hospital Muenster, Germany; 2 Department of Paediatric Rheumatology and Immunology, University Children´s Hospital Muenster, Muenster, Germany; 3 Department of Clinical Chemistry Großhadern, University Munich, Munich, Germany; 4 Department of Pediatric Immunology, University Medical Centre Utrecht, Utrecht, The Netherlands; 5 National Institute of Allergy and Infectious Diseases, National Institute of Health, Bethesda, USA; 6 2nd Division of Pediatrics , "G. Gaslini" Scientific Institute, Genova, Italy; 7 Department of Immunology, Dr. Negrín University Hospital, Las Palmas de Gran Canaria, Spain; 8 Immunology Department-CDB/IDIBAPS, Hospital Clinic, Barcelona, Spain; 9 3rd Department of Pediatrics , Athens University Medical Faculty, Athens, Greece; 10 Service de Génétique Médicale, Centre Hospitalo-Universitaire, Nantes, France; 11 Department of Pediatrics and Neonatology, Nagoya City University, Nagoya, Japan; 12 Department of Gastroenterology, Infectious Diseases and Rheumatology, Charité University Hospital Berlin, Berlin, Germany; 13 Department of Pediatrics, Stanford University Medical Center, Stanford, LA, USA; 14 Division of Clinical Immunology and Allergy, Children's Hospital Los Angeles, LA, USA; 15 Deutsches Zentrum für Kinder- und Jugendrheumatologie, Garmisch-Patenkirchen, Germany; 16 Department of Clinical Chemistry, Charing Cross Hospital, London, UK; 17 Department of Dermatology, University Hospital Muenster, Muenster, Germany; 18 National Institute of Arthritis and Musculoskeletal and Skin Diseases, National Institute of Health, Bethesda, USA

## Introduction

Hyperzincaemia and hypercalprotectinaemia (Hz/Hc), a rare condition within the spectrum of autoinflammatory diseases, is associated with hepatosplenomegaly, arthritis, anemia, cutaneous inflammation, and failure to thrive. So far, no genetic cause has been identified. While the clinical appearance is heterogeneous, all affected individuals present with extremely elevated MRP8/MRP14 (calprotectin) serum concentrations (0.9-12.0 g/l (normal range < 0.001 g/l)).

## Objectives

The clinical phenotype of 12 patients was characterized and compared to 11 patients with classical PAPA syndrome. Screening of candidate genes was performed to identify disease-causing mutations.

## Methods

Serum concentrations of MRP8/14 complex were analyzed in 12 patients with Hz/Hc and compared to 11 PAPA patients. Candidate exons of these patients were sequenced. Cytokine profile of 12 patients with *PSTPIP1* mutations was analyzed by mulitplex ELISA. MRP8/14 secretion from patient´s PBMCs was measured and activity of patient`s sera on monocytes evaluated. The clinical phenotype of all enrolled patients was characterized and compared.

## Results

Ten of twelve patients were heterozygous carriers of a glutamic acid 250 (GAG)→lysine (AAG)/p.Glu250Lys/E250K substitution and 1 patient of a glutamic acid 257 (GAG)→lysine (AAG)/p.Glu250Lys/E257K substitution in exon 11 of the *PSTPIP1* gene. MRP8/MRP14 concentrations were extremely elevated in these patients (0.9-12 g/l) compared to eleven patients presenting with classical PAPA symptoms (0.02-0.35 g/l), whose levels again were significantly higher compared to normal controls. Cytokine profiling confirmed the heterogeneity of *PSTPIP1* mutations with a distinct profile for the Hz/Hc phenotype. MRP8/14 hypersecretion was found in PBMCs of patients with *PSTPIP1* mutations and the serum of patients with active disease showed co-stimulatory properties on monocytes activated with TLR-1 ligands.

## Conclusion

The novel *PSTPIP1* E250K and E275K mutations cause an autoinflammatory disorder known as hyperzincaemia and hypercalprotectinaemia. The disease causes a heterogeneous spectrum of symptoms that only partially overlaps with the presentation of the classical PAPA syndrome. Elevated MRP8/14 levels are a common hallmark and biomarker of disorders caused by mutations in the *PSTPIP1* gene.

## Disclosure of interest

None declared.

